# Delusional Misidentification Syndromes: Untangling Clinical Quandary With the Newer Evidence-Based Approaches

**DOI:** 10.7759/cureus.20165

**Published:** 2021-12-04

**Authors:** Mayank Gupta, Nihit Gupta, Faiza Zubiar, Dhanvendran Ramar

**Affiliations:** 1 Psychiatry, Lake Erie College of Osteopathic Medicine, Erie, USA; 2 Psychiatry and Behavioral Sciences, Clarion Psychiatric Center, Clarion, USA; 3 Psychiatry, University of West Virginia, Glen Dale, USA; 4 Psychiatry, The Trenton Psychiatric Hospital, Trenton, USA; 5 Psychiatry and Behavioral Sciences, Bellin Healthcare, Green Bay, USA

**Keywords:** treatment resistant psychosis, schizophrenia spectrum, capgras, fregoli, delusional misidentification syndromes

## Abstract

The delusional misidentification syndromes (DMS) have been described extensively in the descriptive literature of the last century given its unusual and often-distressing clinical presentations. In the last few decades, there have been advances in scientific research that have identified more precise brain areas involved in these delusional syndromes. Since DMS are reported in both early-onset psychosis and neurodegenerative conditions, the strategies to address and mitigate underlying etiology warrant a thorough assessment and individualized treatment planning. The age of onset, nature of the clinical presentation, the utility of diagnostic tests, and assessment of violence are few among many areas which need attention during clinical management of these rare syndromes.

## Introduction

In the twentieth century, there was an extensive descriptive and phenomenological understanding of delusional misidentification syndromes (DMS) [1]. There are four subtypes of DMS described in the literature: Capgras’ syndrome [2], Frégoli’s syndrome [3-4], the syndrome of intermetamorphosis [5], and the syndrome of subjective doubles [6]. Although these syndromes are observed in clinical practice, they did not find a place in the current nosology of mental health disorders. The majority of these delusions were associated with schizophrenia however, they have also been observed in bipolar disorders and organic brain syndromes [7]. The epidemiological evidence suggests cross-cultural presence of these symptoms but since they are rare the true prevalence is largely unknown [8]. In clinical settings, these symptoms could bring a unique set of challenges given its pervasive nature, highly distressing presentation, diagnostic uncertainty, and medicolegal risks. This case report highlights a few clinical aspects of these issues and discusses management strategies.

## Case presentation

A 20-year-old male was admitted for the third time (in the period of 12 months) to the inpatient unit after the family petitioned an involuntary commitment due to worsening delusions and aggression. Before this admission, he had a history of a psychotic illness and non-adherence with follow-up treatment. After his last discharge from the inpatient hospital, he stopped quetiapine 400mg and as his mental health started deteriorating, he started making threats of harming his parents. He has an insidious onset of symptoms over a period of two years, six months ago dropped out from the university, and a month before the admission refused to identify his father. He also made threats to harm his family, since he believed his real father was now living in Mexico and an imposter had replaced him in the household. The lack of self-care, 20 lbs. weight loss due to fear of being poisoned, anger outbursts, and lack of sleep had all led to a significant decline of his psychotic illness. The family contacted outpatient providers who recommended inpatient care. There was no evidence of any underlying medical condition; all workups including, electroencephalogram and urine toxicology were negative. The bloodwork for hormonal or vitamins dysfunction was also negative. The prior neuroimaging studies including magnetic resonance imaging had no abnormal findings. He had a trial on Lurasidone 40mg, Olanzapine 10mg, and Quetiapine 400mg, however, the response could not be established, because of nonadherence with the treatment. After psychotherapeutic interventions, he agreed to start on a regimen of Paliperidone 3mg monotherapy which was increased to 6mg on day 4 and then 9mg on day 7. He showed some clinical response, evident by improvement in self-care, weight gain, and a brief conversation with his mother on the phone. However, even after two weeks of admission, his response was suboptimal and he continued to have Capgras delusions though admitting that the imposter did not talk like his father. Given, a poor response to three second-generation antipsychotics he agreed to be started on Clozapine augmentation. He was also initiated on Valproic acid 500mg extended-release at night and at 150mg of Clozapine dose, a significant clinical improvement was observed. On this regimen and after four weeks of stay, he was discharged back into the care of the family. The discharge family sessions entailed discussion about the risks of non-adherence, violence, and the importance of follow-up care.

## Discussion

The early onset treatment-resistant schizophrenia has an insidious, serious, and enduring clinical course. In rare instances, they may present with specific delusional misidentification [9]. In most of these cases, the misidentification is of a person with whom they have close emotional ties and often develops ambivalence during clinical manifestations of these symptoms. These presentations prompt the need for evidence-based approaches in assessment, diagnostic battery, and treatment. Besides their descriptive underpinnings of the twentieth century, in the last two decades, and with the advances in neuroscience, there is a more accurate diagnosis and better management of these conditions. DMS are often associated with dementia [10], acquired brain injury, epilepsy, and cerebrovascular accidents. It's widely reported in Alzheimer’s, vascular, Lewy body dementia, and Parkinson’s disease [11]. The neuroimaging studies reveal an association with right hemisphere abnormalities [12], particularly in the frontal and temporal regions [13]. The neuropsychological studies have consistently shown impairments of face processing regions in the brain [14-15]. When the onset of DMS symptoms occurs after the age of 65, it could be suggestive of a degenerative neurocognitive condition. Therefore, a detailed neurological examination and relevant tests including electroencephalogram (EEG), magnetic resonance imaging (MRI) [16-17], or other investigations are an essential part of the clinical workup [18]. There are many case reports of early-onset psychosis with DMS which highlights the risks of violence and homicide. Therefore, it’s imperative to assess for dangerousness [19] and plan interventions to mitigate these risks. Since in most cases, the imposters replace the family members, therefore, parents, siblings, and spouses remain at risk. The evidence of Clozapine in early-onset treatment-resistant schizophrenia is robust and is recommended choice of drug as monotherapy or augmentation agent [20]. The age of onset of these symptoms may guide us to assert its nosology and help to develop an appropriate diagnostic workup to establish an etiologic basis of DMS. In absence of an identifiable neurological etiology, the treatment plan must target impairments due to the underlying psychotic illness. Given the refractory nature of these disorders, very close monitoring is essential to address frequent nonadherence with treatment. There is substantial evidence of associated aggression, and violence, therefore psychoeducation of the patients and families is imperative to mitigate the medicolegal risks associated with these conditions.

## Conclusions

The DMS is a complex range of symptoms overlapping in various psychiatric and neurological disorders. It has been extensively described in the older literature but not been included in the modern manualized nosology and classification of mental disorders. However, DMS continues to make rounds in the realm of clinical practice. Therefore, knowledge of its unique and myriad presentations in absence of clear etiologic understandings is critical, and sufficient recent evidence is there to address these clinical challenges. The earlier onset of these symptoms in absence of organic brain disorder points towards the serious psychiatric condition and therefore robust interventions to manage the risks are warranted. Besides the etiological conundrums of DMS, the assessment of associated violence and aggression is imperative and must be part of the treatment planning and management.
